# Comparative Susceptibility of Plasmodium ovale and Plasmodium falciparum Field Isolates to Reference and Lead Candidate Antimalarial Drugs in Ghana

**DOI:** 10.1128/spectrum.04916-22

**Published:** 2023-04-24

**Authors:** Yaw Aniweh, Alamissa Soulama, Jersley Chirawurah, Felix Ansah, Harry A. Danwonno, Fanta Sogore, Melanie Rouillier, Brice Campo, Lucas Amenga-Etego, Abdoulaye A. Djimde, Gordon A. Awandare, Laurent Dembele

**Affiliations:** a West African Centre for Cell Biology of Infectious Pathogens (WACCBIP), College of Basic and Applied Sciences, University of Ghana, Legon, Ghana; b Malaria Research and Training Centre (MRTC), Faculty of Pharmacy, Université des Sciences, des Techniques et des Technologies de Bamako (USTTB), Point G, Bamako, Mali; c Medicines for Malaria Venture (MMV), Geneva, Switzerland; University of Huddersfield

**Keywords:** *Plasmodium ovale*, antimalarial drug susceptibility testing, field isolates

## Abstract

Malaria treatments resulted in the decline of the deadliest Plasmodium falciparum globally while species, such as *P. ovale*, infections have been increasingly detected across sub-Saharan Africa. Currently, no experimental drug sensitivity data are available to guide effective treatment and management of *P. ovale* infections, which is necessary for effective malaria elimination. We conducted a prospective study to evaluate *P. ovale* epidemiology over 1 year and determined *ex vivo* susceptibility of the field isolates to existing and lead advanced discovery antimalarial drugs. We report that while P. falciparum dominated both symptomatic and asymptomatic malaria cases, *P. ovale* in mono or co-infections caused 7.16% of symptomatic malaria. Frontline antimalarials artesunate and lumefantrine inhibited *P. ovale* as potently as P. falciparum. Chloroquine, which has been withdrawn in Ghana, was also highly inhibitory against both *P. ovale* and P. falciparum. In addition, *P. ovale* and P. falciparum displayed high susceptibility to quinine, comparable to levels observed with chloroquine. Pyrimethamine, which is a major drug for disease massive prevention, also showed great inhibition of *P. ovale*, comparable to effects on P. falciparum. Furthermore, we identified strong inhibition of *P. ovale* using GNF179, a close analogue of KAF156 imidazolopiperazines, which is a novel class of antimalarial drugs currently in clinical phase II testing. We further demonstrated that the *Plasmodium* phosphatidylinositol-4-OH kinase (PI4K)-specific inhibitor, KDU691, is highly inhibitory against *P. ovale* and P. falciparum field isolates. Our data indicated that existing and lead advanced discovery antimalarial drugs are suitable for the treatment of *P. ovale* infections in Ghana.

**IMPORTANCE** Current malaria control and elimination tools such as drug treatments are not specifically targeting *P.ovale*. *P. ovale* can form hypnozoite and cause relapsing malaria. *P. ovale* is the third most dominant species in Africa and requires radical cure treatment given that it can form liver dormant forms called hypnozoites that escape all safe treatments. The inappropriate treatment of *P. ovale* would sustain its transmission in Africa where the medical need is the greatest. This is a hurdle for successful malaria control and elimination. Here, we provided experiment data that were lacking to guide *P. ovale* treatment and disease control policy makers using reference antimalarial drugs. We also provided key experimental data for 2 clinical candidate drugs that can be used for prioritization selection of lead candidate’s identification for clinical development.

## INTRODUCTION

Plasmodium ovale, discovered in 1922 in the blood of a soldier returning from East Africa (1), is one of the least studied of the 5 malaria parasites that infect humans. For many years it had been frequently confused with P. vivax. However, the recent improvement in malaria diagnosis (2) has shown that *P. ovale* is widespread in sub-Saharan Africa (3–8), in Myanmar (9), the eastern parts of Indonesia and the Philippines (10). Thus, although *P. ovale* was first described as an infectious disease of humans by Stevens in 1922, there are still large gaps in our knowledge of the importance and significance of its infections as causes of malaria in sub-Saharan Africa.

To prevent mortality and minimize morbidity caused by *P. ovale*, prompt and accurate diagnosis and treatment are necessary. For *P. ovale* infections treatment, the USA Centers for Disease Control (USA CDC) had suggested chloroquine (https://www.cdc.gov/malaria/resources/pdf/treatment_guidelines_101819.pdf). The world health organization (WHO) recommends the use of a combination of primaquine with either chloroquine or with artemisinin combination therapy to enable radical cure of this parasite (https://apps.who.int/iris/bitstream/handle/10665/162441/9789241549127_eng.pdf;jsessionid=45A93C749DB269256A80888D59C6A596?sequence=1). However, chloroquine has been withdrawn in sub-Saharan Africa because of P. falciparum resistance (11). Also, primaquine causes hemolysis anemia in glucose-6- phosphate dehydrogenase (G6PD) deficient patients. G6PD deficiency is high in Sub-Saharan Africa (12, 13). Current case reports and studies have shown the additional great concern of *P. ovale* reduced susceptibility to frontline ACTs (6, 14, 15). Thus, there is an urgent need for data on *P. ovale* drug susceptibility to guide efficient treatment and management of its infections. As the current global goal is to eliminate malaria, *P. ovale* would be a reservoir that will compromise achievement of malaria elimination strategies if treatment and management of its infections remain ineffective.

Herein, we conducted a prospective study assessing the epidemiology of P. falciparum, *P. malariae*, and *P. ovale* infections over 1 year in Central, Bono east and Eastern regions of Ghana in 2019 (7). Along with the study, we evaluated the susceptibility of *P. ovale* isolates freshly collected from the field to reference antimalarial drugs approved for malaria treatment. Furthermore, we tested the potential of lead advanced discovery antimalarial drug candidates, including GNF179 (16), a close analogue of KAF156 imidazolopiperazine, which is a novel class of antimalarial drug currently in clinical Phase IIb trial. Finally, we also tested the *Plasmodium* PI4K-specific inhibitor KDU691 (17) against *P. ovale* and P. falciparum.

## RESULTS

### P. ovale caused symptomatic malaria and has no compromised susceptibility to reference antimalarials *ex vivo*.

**FIG 1:**
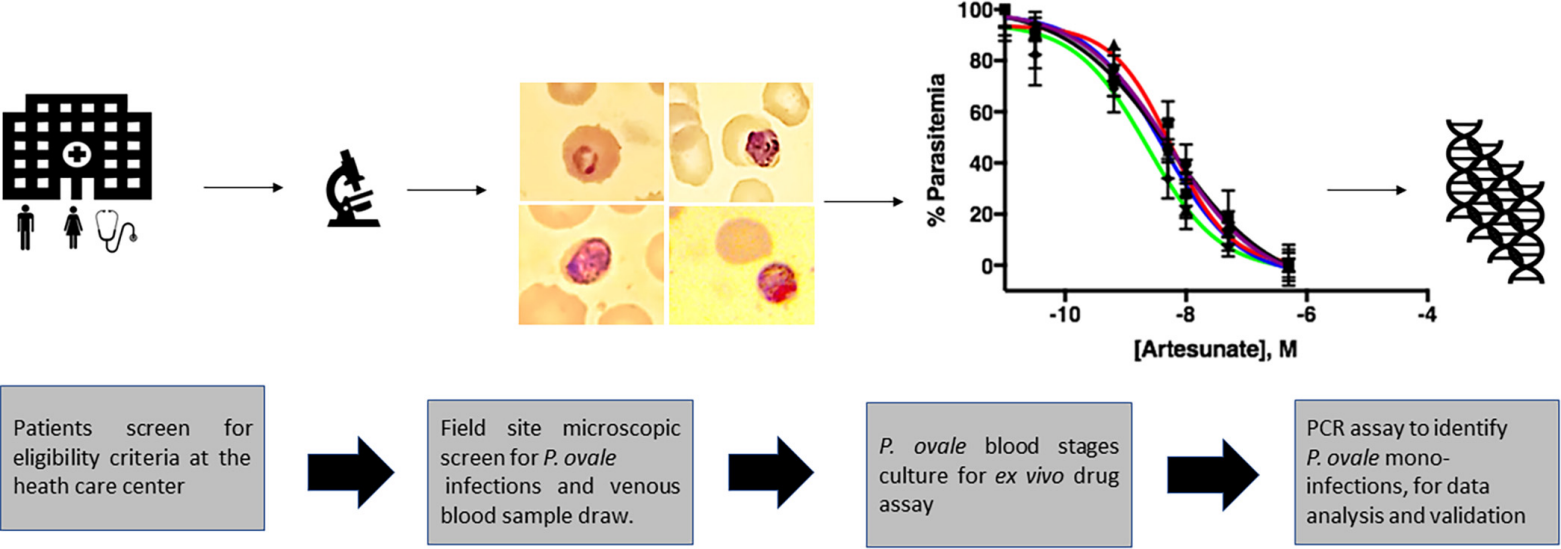
Sample collection, drug, and PCR assays workflow design.

This study was conducted as displayed in Fig. 1. The participants in this study were screened for symptomatic and asymptomatic malaria caused by the different *Plasmodium* species. Generally, it was observed that P. falciparum was the most dominant species causing malaria, followed by *P. malariae* and *P. ovale* (Table 1). Among symptomatic malaria, *P. ovale* contributed to 7.16% in mono and mixed infections (Table 1). While the symptoms associated with its infection are thought to be benign, mono infections of *P. ovale* represented 2.1% (*N* = 27) in symptomatic participants and 0.54% (*N* = 7) in asymptomatic participants (Table 1). Thus, *P. ovale* caused more symptomatic malaria than asymptomatic malaria in the participants studied, indicating the need to consider screening drugs against *P. ovale*.

**TABLE 1:** Epidemiology of P. falciparum, *P. malariae*, and *P. ovale* infections over 1 year in symptomatic and asymptomatic participants in Central, Bono east and Eastern regions of Ghana in 2019^*a*^

*Plasmodium* species		Pf	Pm	Po	Pf+Pm	Pf+Po	Pm+Po	Pf+Pm+Po	All species
Symptomatic Patients (*N* = 1608)	Prevalence (%)	63.12	3.3	1.68	7.84	3.73	0.06	0.25	79.98
	Frequency (%)	78.93 (*N* = 1,015)	4.12 (*N* = 53)	2.1 (*N* = 27)	9.80 (*N* = 126)	4.67 (*N* = 60)	0.08 (*N* = 1)	0.31 (*N* = 4)	100 (*N* = 1286)
Asymptomatic patients (*N* = 1640)	Prevalence (%)	23.35	0.3	0.43	0.91	0.49	0	0	25.37
	Frequency (%)	92.07 (*N* = 383)	1.20 (*N* = 5)	0.54 (*N* = 7)	1.17 (*N* = 15)	0.62 (*N* = 8)	0 (*N* = 0)	0 (*N* = 0)	100 (*N* = 416)

a We calculated prevalence and frequency as follows: The prevalence is the number of malaria positive cases among the whole population screened while the frequency is the number of each specie among all species or all positive malaria cases. Prevalence = (number of malaria positive cases/number of all participants) x 100. Frequency of *P. ovale* = (number of *P. ovale* cases/number of all species detected malaria cases) x 100.

**FIG 2:**
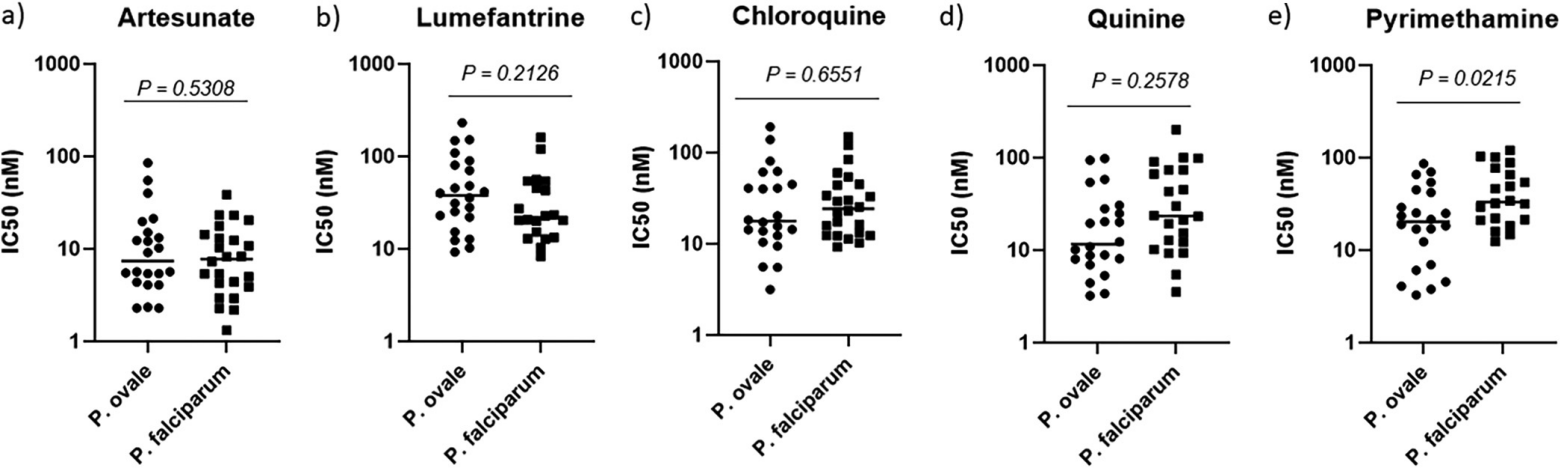
Plasmodium ovale and Plasmodium falciparum field isolates comparative susceptibility to reference approved antimalarial drugs: a) artesunate, b) lumefantrine, c) chloroquine, d) quinine, and e) pyrimethamine.

Having established the epidemiology of *P. ovale* infections and their importance in symptomatic malaria, we evaluated the *ex vivo* susceptibility of *P. ovale* to a panel of currently approved antimalarial drugs at Kintampo north-Ghana in 2019. This included artesunate, lumefantrine, chloroquine, quinine, and pyrimethamine (Fig. 2). Artesunate or lumefantrine (in combination with artemether), that constitutes the current recommended treatment for any *Plasmodium* species infections in Ghana, potently inhibited *P. ovale* like P. falciparum (Fig. 2a and b). Z' factors value was an important criterion to optimize and validate the *P. ovale* assay. We aimed to have similar Z' factors value for both P. falciparum and *P. ovale*. In this regard, using parasitemia below 0.5% yielded mainly poor Z' factors. Thus, only optimal 0.5% parasitemia of *P. ovale* was used and our average Z' factor obtained for P. falciparum assay using 12 isolates was 0.81 while Z’ for 12 isolates of the *P. ovale* assay was 0.68. No Z’ factors value below 0.5 was included in the data used in this current manuscript. No difference was observed in the inhibitory concentrations 50% (IC_50_) median of artesunate against *P. ovale* (7.39 nM) when compared to the one against P. falciparum (7.76 nM) (Table 2). Similarly, lumefantrine displayed no difference in its IC_50_ median against *P. ovale* (37.92 nM) when compared to P. falciparum (21.72 nM) (Table 2). Chloroquine, that has been withdrawn from Ghana because of P. falciparum drug resistance, potently inhibited *P. ovale* like P. falciparum at the nanomolar range with IC_50_ medians of 17.84 nM and 24.35 nM, respectively (Fig. 2c and Table 2) excepted for 2 isolates of each specie that displayed IC50s > 100 nM (Fig. 2c). Thus, in this study a decreased susceptibility to chloroquine was observed for both *P. ovale* and P. falciparum (Fig. 2c). Furthermore, we included quinine that has been historically used as an efficient malaria treatment, as well as pyrimethamine that is part of the disease chemoprevention treatment (Fig. 2d and e). Both these compounds displayed potent inhibition of *P. ovale* like P. falciparum (Fig. 2d and e). Thus, our data indicated that *P. ovale* parasites can be treated as efficiently as P. falciparum, even with chloroquine that is not recommended in Ghana for malaria treatment anymore because of P. falciparum resistance.

**TABLE 2:** Fifty percent inhibitory concentration (IC_50_) of reference approved antimalarial drugs artesunate (ART), lumefantrine (LUM), chloroquine (CA), quinin (QN), and pyrimethamine (Pyr) against Plasmodium ovale and Plasmodium falciparum field isolates

	IC_50_ (nM) of reference antimalarial drugs against *P. ovale* and P. falciparum clinical field isolates									
	*P. ovale* field isolates					P. falciparum field isolates				
Compounds	CQ	ART	LUM	Pyr	QN	CQ	ART	LUM	Pyr	QN
No. of isolates tested	22	22	22	22	22	22	22	18	18	20
Median IC50 (nM)	17.84	7.39	37.92	20.3	11.6	24.35	7.76	21.72	33.17	23.42
Mean IC50 (nM)	39.29	15.50	58.32	27.29	24.5	36.94	10.32	38.08	47.98	44.98
Range IC50 (nM)	3.18 to 192.7	2.3 to 85.16	9.26 to 152.07	3.29 to 86.39	3.22 to 98.03	9.27 to 150.09	2.19 to 38.5	8.34 to 160.33	12.36 to 120.76	3.54 to 201.45

### Lead advanced discovery antimalarial drug candidates GNF179 imidazolopiperazine and the *Plasmodium* PI4K-specific inhibitor KDU691 potently inhibited *P. ovale* and P. falciparum.

**FIG 3:**
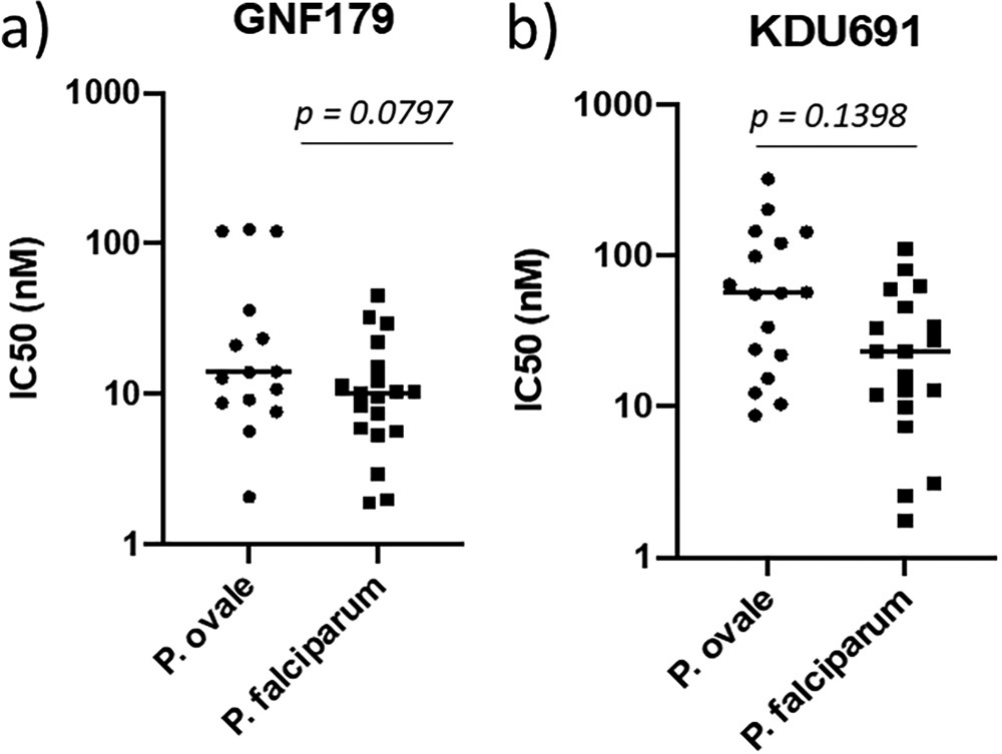
Plasmodium ovale and Plasmodium falciparum field isolates comparative susceptibility to antimalarial drug candidates: a) imidazolopiperazines (IPZ) GNF179 and b) *Plasmodium* phosphatidylinositol-4-OH kinase (PI4K)-specific inhibitor KDU69.

Having shown that *P. ovale* is susceptible to the approved reference antimalarial drugs for malaria treatment, we assessed the potential of lead advanced discovery antimalarial drug candidates to treat *P. ovale* (Fig. 3). This included GNF179, a close analogue of the imidazolopiperazine KAF156 that is currently in phase II clinical trial in Africa and Asia. GNF179 showed a strong inhibition of P. falciparum as expected, as well as a strong inhibition of *P. ovale* (Fig. 3a) with IC_50_ medians of 14 nM and 10.22 nM, respectively (Table 3). The *Plasmodium* PI4K-specific inhibitor KDU691 also potently inhibited both *P. ovale* (56.34 nM) and P. falciparum (23.19 nM) (Fig. 3b and Table 3). Thus, these new classes of antimalarial drug candidates would be excellent tools to treat malaria caused by not only P. falciparum infections but also *P. ovale* infections if they were approved for disease treatment.

**TABLE 3:** Fifty per cent inhibitory concentration (IC_50_) of antimalarial drug candidates imidazolopiperazines (IPZ) GNF179 and the *Plasmodium* phosphatidylinositol-4-OH kinase (PI4K)-specific inhibitor KDU691 against Plasmodium ovale and Plasmodium falciparum field isolates

IC_50_ (nM) of candidate antimalarial drugs against *P. ovale* and P. falciparum clinical field isolates				
Parasite species	*P. ovale* field isolates		P. falciparum field isolates	
Compounds	KDU691	GNF179	KDU691	GNF179
Isolates tested	17	15	19	19
Median IC50 (nM)	56.34	14	23.19	10.22
Mean IC50 (nM)	81.68	35.37	30.33	13.04
Range IC50 (nM)	8.72 to 320.12	2.07 to 124.23	1.74 to 110.33	1.89 to 45.23

## DISCUSSION

Antimalarial drugs for global control and elimination are identified and developed to mainly treat P. falciparum infections. These compounds do not necessarily target *P. ovale* and the other *Plasmodium* species. Similarly, to what is observed for treatments, the current surveillance and diagnostic tools are designed mainly for P. falciparum. Thus, *P. ovale* is being neglected in malaria control efforts specifically in the process of antimalarial drug discovery and development that is an important component of the elimination strategy. This results in critical shortage of data on *P. ovale*’s epidemiology and susceptibility to antimalarial drugs. In this study, we screened participants for all *Plasmodium* species and established their epidemiology, as well as the comparative susceptibility of *P. ovale* and P. falciparum resulting field isolates to a panel of reference antimalarial drugs already approved for malaria treatment and drug candidates being developed to treat malaria.

When screened for all *Plasmodium* species, P. falciparum appeared as the most frequent and prevalent malaria parasite specie, followed by *P. malariae* and *P. ovale* (Table 1) as reported earlier in Ghana (7, 18).

Through the establishment of an optimal assay for *P. ovale*, we faced the challenge of low parasitemia infections of this parasite and difficulty to generate a good Z’ factor value. Thus, if the P. falciparum assays were started also at 0.5% parasitemia (instead of 1%), perhaps these P. falciparum Z' factors would be similar to that of *P. ovale*. However, getting higher *P. ovale* parasitemia (and possibly better Z' factors) is challenging as these are generally low parasitemia infections.

*P. ovale* is a P. vivax-like parasite which treatment remains more challenging than the most prevalent P. falciparum species. The 2 drugs composing the lead treatment currently in use in Ghana for P. falciparum malaria, artesunate and lumefantrine (in combination with artemether), were shown to potently inhibit both P. falciparum and *P. ovale* field isolates from Ghana (Fig. 2). Some *in vivo* studies have however suggested that artemether and lumefantrine combined have suboptimal efficacy against some *P. ovale* field isolates in Ghana (14) and in Mali (15, 19). Looking at the individual IC50s obtained in this study for artesunate and lumefantrine, the distribution is quite large for both P. falciparum and *P. ovale*, with some isolates displaying IC50s more than 5-fold higher than the IC50 median. These higher IC50s could result from a decreased susceptibility to these drugs. Parasites genotyping along with clinical studies on *P. ovale* treatment efficacy would be the way forward to answer the question of preexisting resistance and treatment failure. Whole genome sequencing of *P. ovale* isolates with a wide spread of susceptibility to particular antimalarial agents would be necessary to demonstrate the possibility of resistance to these agents and to develop genotyping protocols. We believe this would be a great resource or addition to the already existing data.

Like artesunate and lumefantrine, chloroquine displayed a strong inhibition of *P. ovale* and P. falciparum fields isolates (Fig. 2 and Table 2). Chloroquine has been withdrawn in Ghana and many other sub-Saharan African countries because of P. falciparum drug resistance to this antimalarial. A larger study coupled with molecular data would be needed in defining Chloroquine’s suitability for treating co-infections amid the current withdrawal from the clinics in Africa (20, 21). Quinine, another old important antimalarial drug for almost 400 years, also potently inhibited *P. ovale* and P. falciparum fields isolates (Fig. 2 and Table 2). A previous report suggested that quinine continued use is challenged by its poor tolerability and compliance with complex dosing regimens (22). Finally, as last reference antimalarial we tested pyrimethamine that showed nanomolar potency against *P. ovale* and P. falciparum fields isolates (Fig. 2 and Table 2). Pyrimethamine is currently used in combination with sulfadoxine for the chemoprevention of malaria. Thus, most of the reference compounds evaluated herein are readily available to efficiently treat *P. ovale* infections. Our data can thus help malaria control program in the use of the reference antimalarial to treat *P. ovale* malaria.

As extensive efforts are ongoing to discover and develop next generation antimalarial drugs to eliminate malaria, we tested 2 lead advanced discovery candidate antimalarial drugs. The imidazolopiperazines (IPZ) GNF179 and the *Plasmodium* phosphatidylinositol-4-OH kinase (PI4K)-specific inhibitor KDU691, which are both novel class of antimalarial drug currently in clinical development. Both GNF179 and KDU691 demonstrated strong inhibitory activity against *P. ovale* and P. falciparum field isolates (Fig. 3 and Table 3). If approved for malaria treatment, these classes of antimalarial drugs would be valuable to treat diverse *Plasmodium* species (23, 24).

## MATERIALS AND METHODS

### Ethical considerations.

The current study protocol was reviewed and approved by the Ghana Health Service Ethical Review Committee of Ghana with the reference GHS-ERC:005/12/17. Only participants or their parent/guardian who provided written informed consent, plus children able to understand the study and who gave assent were enrolled in this study. All patients with malaria that consented to participate in the study were enrolled and treated using recommended artemether-lumefantrine (AL) to clear the parasites.

### Study design and site population screen.

We conducted a cross-sectional screening and detection of all *Plasmodium* malaria cases during a longitudinal prospective study aimed at assessing *ex vivo* efficacy of panel of antimalarials against *P. ovale*. All field isolates were obtained from Volta, Central, Bono east, and Eastern regions of Ghana in 2019 from January 01^st^ to December 31^st^. We collected field isolates of *P. ovale* parasites that were freshly used for drug assays while PCR assays were conducted to detect *P. ovale* mono-infection samples from other *Plasmodium* species infection samples. This enabled the further validation of assay data done with mono infection samples.

### Laboratory procedures and data analysis.

**(i) PCR assay.** Genomic DNA from the patient’s samples were extracted from dried blood spots using the QIAamp DNA minikit only (Qiagen) following the manufacturer’s instruction. PCR assay was conducted for *Plasmodium* species differentiation using the small subunit rRNA 18s (ssrRNA) gene (25). Further *Plasmodium* speciation was done using both modified cooperative primers and conventional primers described by Ansah et al. (7). The entire procedure has been reported by Ansah et al. (7).

**(ii) *Ex vivo* drug assay.** Following *Plasmodium* species confirmation by microscopy and PCR, the parasite samples were washed and prepared for *ex vivo* drug screening. Compounds tested included artesunate (A3731-100MG), lumefantrine (L5420), chloroquine (PHR1258-1G), quinine (22620), and pyrimethamine (46706) as reference compounds; the GNF179, a close analogue of KAF156 imidazolopiperazines (IPZ), and the *Plasmodium* phosphatidylinositol-4-OH kinase (PI4K)-specific inhibitor KDU691 as clinical candidates provided by Novartis. For the *ex vivo* drug assay, 3 to 6 h freshly collected venous blood samples containing at least 80% ring stage *P. ovale* and/or P. falciparum asexual stages were used for parasite cultivation and antimalarial screening. P. falciparum and *P. ovale* clinical isolates were cultured as per standard protocol of P. falciparum. In brief, isolates were washed in RMPI1640 media three times and diluted to 1% and 0.5% parasitemia, respectively, for P. falciparum and *P. ovale*. The diluted parasites were dispensed into 96-well plates. The set-up was maintained at 37° C in RPMI 1640 medium (Sigma) supplemented with 0.5% healthy individual plasma, 2 mg/mL sodium bicarbonate, 50 μg/mL gentamicin (Sigma), and 2% AB^+^ heat-inactivated normal human serum (PAN Biotech). It was observed earlier during the optimization of the protocol for the assays that we needed to supplement with plasma before we could measure the increase in the DNA content after the 48 h of incubation. Since the patient direct plasma may have possible antibodies or other components that may inhibit growth, we obtained healthy individual plasma from the same community and supplemented the media. All cultures were adjusted to 4% hematocrit using O^+^ erythrocytes from a single donor and incubated in an atmosphere of 2% O_2,_ 5% CO_2_, and balanced with nitrogen gas. P. falciparum culture conditions were used for *P. ovale* cultivation, in addition to further supplementation with patient plasma.

Ten (10 mM) dimethyl sulfoxide (DMSO) stock compounds were 1/3 serial diluted starting at 10 μM into 8 concentration points and tested in duplicated wells. At least 15 independent isolates were tested against each compound. The parasitemia corresponding to each culture well after 48 hours of incubation was determined by replacing 80 μL of the supernatant with SYBR Green I (Invitrogen) stain in lysis buffer (20 mM Tris [pH 7.5], 5 mm EDTA, 0.008% [W/V] saponin, and 0.08% [V/V] Triton X-100) in 1X final concentration, based on established methods (16). The plates were then incubated at 37°C in the dark for at least 30 min, and the total fluorescence from each of the wells was determined with the Varioskan Lux multimode microplate fluorescent plate reader (ThermoFisher Scientific) at an excitation of 485 nm, emission of 520 nM, and with a gain of 100. Fluorescence data were plotted using GraphPad Prism 8. The data are curve fit with a variable slope function to estimate IC_50_ values. For each isolate, a Z’ factor to assess assay quality was calculated from positive controls (8 drug-free wells) and negative controls (8 parasite-free, red blood cell control wells). Z’ values over 0.5 are considered good assays, but each curve is examined by eye for suitability. Some assays with Z’ below 0.5 may be considered valid depending on factors, such as the standard error of the curve fit IC_50_. Dose-response curves and IC_50_ were calculated by non-linear regression analysis using GraphPad Prism software version 8 with the data previously normalized to the untreated controls. Statistical tests were done using GraphPad Prism software version 9 and nonparametric Mann-Whitney-U test. A *P* value <0.05 was considered significant.
